# Association between FTO gene polymorphism and obesity in down syndrome children

**DOI:** 10.1007/s00431-024-05909-5

**Published:** 2024-12-21

**Authors:** Shereen A. Mourad, Reham M El-Farahaty, Mohamed A. Atwa, Sohier Yahia, Abdel-Hady El-Gilany, Ahmed A. Elzeiny, Eman S. Elhennawy

**Affiliations:** 1 Department of Clinical Pathology Faculty of Medicine, Mansoura University, Mansoura, Egypt; 2 Department of Pediatrics Genetics Unit Faculty of Medicine, Mansoura University, Mansoura, Egypt; 3 Public Health Department Faculty of Medicine, Mansoura University, Mansoura, Egypt; 4 Lecturer in Department of Clinical Pathology Mansoura Faculty of Medicine, Mansoura University, Mansoura, Egypt

**Keywords:** Down syndrome, Fat mass and obesity associated gene, FTO, Rs17817449, Obesity

## Abstract

Children with Down syndrome (DS) have a higher incidence of overweight and obesity compared to typically developing peers. The fat mass and obesity-associated gene (FTO) is one of the early identified genes linked to obesity in various populations. To date, the FTO rs17817449 gene polymorphism has not been investigated in overweight/obese-DS (ODS) individuals. The current study aimed to explore the potential association between the FTO rs17817449 gene polymorphism and obesity-related markers, and to evaluate the ability of this polymorphism in the prediction of overweight/obesity in DS children and adolescents. This case-control study included 100 DS children under the age of 18, classified into three groups according to BMI-percentile; 50 non-obese DS (NODS), 24 overweight DS, and 26 ODS. Genotyping of FTO gene rs17817449 polymorphism was performed using the restriction fragment length polymorphism (RFLP-PCR) method. Serum lipid and thyroid profiles were also assessed. The results revealed significant increase in the frequency of the FTO rs17817449 T allele among overweight /ODS children compared to NODS children (p=0.0099). Overweight/ODS children exhibited significantly higher frequencies of the FTO rs17817449 GT and TT genotypes compared to NODS children.

*Conclusion*:There is an association between FTO rs17817449 genetic variant and overweight/obesity among the studied DS groups. The FTO rs17817449 GT and TT genotypes, as well as TGs level, were identified as independent risk factors for predicting overweight and obesity in DS children.

**Table:** 

**What is Known:**	
• *Overweight and obese-DS (ODS) children displayed higher BMI and atherogenic lipid profile than non-obese DS children (NODS). FTO gene polymorphism rs17817449 contributes to obesity development in general population, but there is conflicting information about the risk allele*.	
**What is New:**	
• *FTO rs17817449 TT genotype and T allele were considered as independent risk factors for overweight and obesity development in DS children, so they could be used for obesity prediction in DS children*.

**Supplementary Information:**

The online version contains supplementary material available at 10.1007/s00431-024-05909-5.

## Introduction

 Down syndrome, caused by an additional chromosome 21 [1], is considered one of the most frequent chromosomal diseases in children resulting in intellectual disability [2]. The incidence of DS in Egypt is 1∶555 and 1∶770 live births [3–5]. DS individuals had higher rates of obesity compared to the non-DS ones. Many factors contribute to the development of obesity in DS as high serum leptin levels [6, 7], genetic susceptibility, hypothyroidism, low physical activity, increased serum lipids, and an abnormal diet. Other factors as decreased muscle tone, low metabolic rate, depression, low social and financial support, decreased intellectual function could precipitate also to the obesity development in DS because they could affect physical activity and type of food [8]. Obesity in DS patients increases their susceptibility for dyslipidaemia, hyperinsulinemia, obstructive sleep apnoea, certain types of malignancies and abnormal gait [9]. Furthermore, obesity may impair the care of these individuals, and affect their quality of life [10].

Obesity is a complex multifactorial disorder [8] that results from the interaction between environmental factors and obesity-associated genes [11]. Many genetic elements contribute to the development of obesity [12]. FTO was the first gene that was associated with body mass index (BMI) and the risk of obesity [13, 14]. Hypothalamic nuclei [15], which are responsible for energy intake and energy consumption, express FTO mRNA. FTO acts primarily as a demethylase and controls energy balance, adipogenesis and methylation of DNA [16]. Previous studies found a significant relation between lifestyle factors as the overconsumption of fat rich diet and refined starches [17] and sedentary lifestyle with FTO overexpression [18, 19]. Numerous SNPs of the FTO gene were investigated for their possible role in obesity; as rs178117449, rs9939609 rs3751812, rs1421085, rs9930506 (has the greatest effect on body weight and composition), and rs7202116 [16]. These SNPs are located within a 47 kb linkage disequilibrium (LD) block involving segments of the first two introns and exons 2 of FTO. The proximity of the retinitis pigmentosa GTPase regulator interacting protein-1like (RPGRIP1L) gene transcription site and the the 5’ end of FTO [20], may affect RPGRIP1L expression in the brain via FTO genetic polymorphisms, leading to obesity development by influencing leptin signalling [21], and increasing its secretion [6]. A recent meta-analysis revealed that the FTO (rs9939609, rs1421085, rs1861868, rs1477196 & rs17817449) variants were assessed in obese children, while FTO rs9939609 and rs9935401 were examined in overweight and obese children. However, only FTO rs6499640 was evaluated in overweight children, but revealed nonsignificant association [22]. Up till now, the studied FTO rs17817449 variant isn’t evaluated in overweight children and adolescents.

Individuals carrying the FTO rs178117449 risk allele have been found to have an increased risk of obesity [16, 23–25]. However, there is controversy surrounding the risk allele of FTO rs17817449 linked to obesity [12, 26]. Due to these controversies, additional studies are needed to confirm the risk allele of the FTO rs17817449 SNP associated with obesity. To the best of our knowledge, this is the first study to evaluate the efficiency of the FTO rs17817449 polymorphism in predicting overweight and obesity in a cohort of Egyptian DS children.

## Materials and methods

The current case-control study was performed on 100 DS children confirmed by cytogenetic analysis. These children were recruited from the genetic unit of Mansoura University Children Hospital. They were divided according to BMI-centile into three groups: NODS: 50 children (29 males 58% and 21 females 42%), aged from 3.5 to 16 years, their BMI percentiles were > 5th and < 85th. The second group consisted of 24 overweight children (12 males (50%) and 12 females (50%), aged from 3.5 to 16 years, their BMI percentiles were ≥ 85th and < 95th centile. The third group included ODS children (16 males (61.5%) and 10 females (38.5%), aged 3.5–14 years. Their BMI-centile was ≥ 95th centile. DS children with hypothyroidism, congenital heart diseases or receiving any medications known to affect growth as growth hormone were excluded from the study.

Medical history, clinical examination, and anthropometric measurements were performed. Waist (WC), mid-arm (MAC), head and hip circumferences (HC) were measured in cm. BMI was calculated as the ratio of weight in kg to the height in m².

A fasting serum blood sample was collected to assess the lipid profile, including total cholesterol (TC), triglycerides (TG), and high-density lipoprotein (HDL-C), using a Cobas C311 (Roche Hitachi) analyzer. Low-density lipoprotein (LDL-C) was calculated using Friedewald formula. Serum T3, T4 and TSH were estimated using a Cobas e411 (Roche Diagnostics) analyzer.

DNA samples were extracted from 3 ml of venous blood, collected from all participants in EDTA tubes, using G-spin total DNA Extraction Kit (Intron Biotechnology). FTO SNP genotyping was performed using PCR-RFLP method. Amplification of the region containing the FTO SNP was done according to Scott et al. [27], , using a set of 2 primers:

Forward 5′-CGGTGAAGAGGAGGAGATTG-3′.

Reverse 5′-CATCTCTGCCCCAGTTTCTC − 3′.

PCR amplification was performed using 2x PCR Master Mix solution (i-Taq TM) iNtRON Biotechnology, and a thermal cycle (Bio-Rad PTC-100 peltier, USA). The thermal cycling conditions were as follows as recommended by Zermeño-Rivera et al. [28], : initial denaturation at 95ºc for 5 min, followed by 35 cycles of denaturation at 94ºc for 30 s, annealing at 57ºc for 30 s, extension at 72ºc for 30 s, and a final extension at 73ºc for 2 min.

**Fig. 1:**
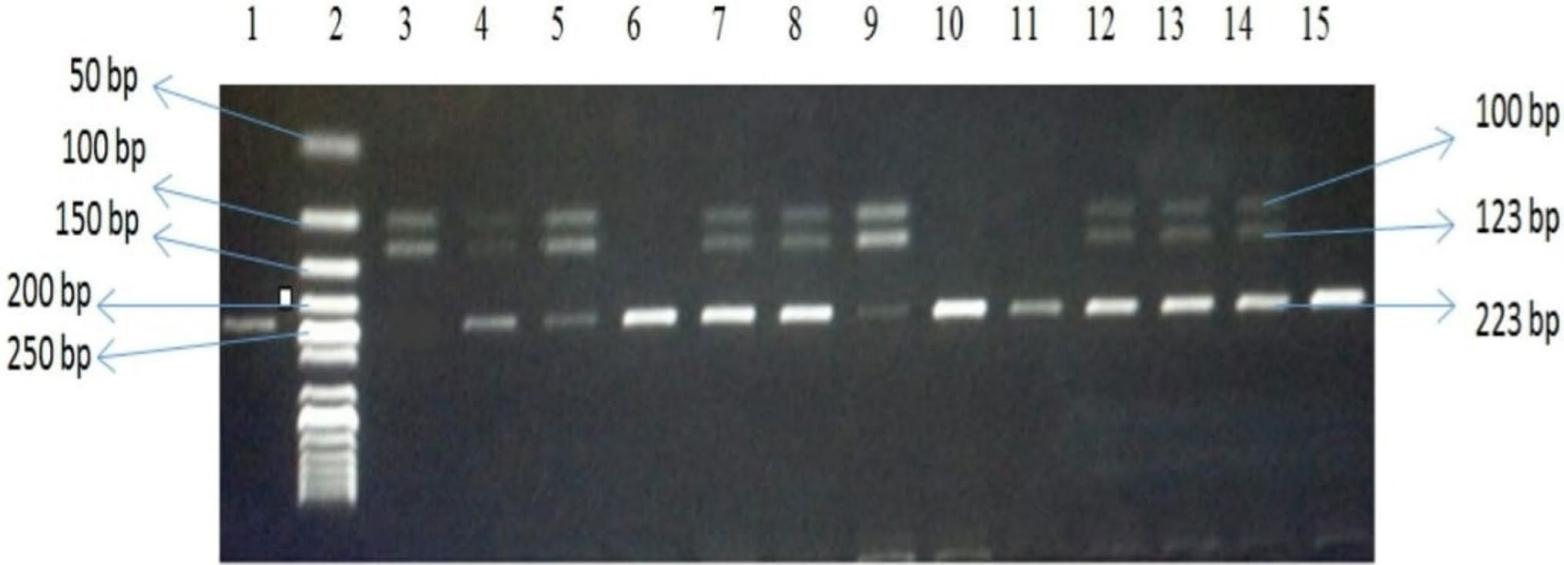
A micrograph of 2.5% agarose gel electrophoresis showing restriction fragments of FTO rs17817449, Lane 2 shows 50 bp DNA ladder (reference bands 50,100,150, 200, 250 bp). Lanes 1, 6, 10, 11, 15 show homozygous wild genotype GG (223 bp). Lane 3 shows homozygous mutant genotype TT (100, 123) while Lane 4, 5, 7, 8,9,12, 13, 14 show heterozygous genotype GT (100, 123, 223 bp)

The amplified PCR products (223 bp for rs17817449) were digested with restriction enzyme *AlwNI* (Thermo scientific, lot no. 00474696), and loaded on 2.5% agarose gel and stained with ethidium bromide against a 50 bp ladder (Thermo Scientific, lot. No. SM0373). The restriction fragments were: 223 bp band for wild type (GG), 100 and 123 bp bands for homozygous (TT), and 223, 100, 123 bp bands for heterozygous (TG) genotypes (Fig. 1).

### Statistical analysis

The data were analysed using the Statistical Package for Social Science (IBM Corp. Released 2011.IBM SPSS Statistics for Windows, Version 24.0. Armonk, NY: IBM Corp). Mean and standard deviation (± SD) were used for the parametric numerical data while for non-parametric numerical data, median and min-max were used. Frequency and percentage were used for categorical data. To assess the significant difference between two group means, student’s *t* test was used. One way ANOVA test was utilized to compare means for more than two groups. Mann-Whitney U test was utilized to assess the statistical difference between the medians of two groups´ and Kruskal Wallis test to compare the median of more than two groups. To examine the relationship between two or more non-numerical variables, Chi-Square test was utilized. Logistic regression analysis was performed for prediction of risk factors. Odds ratio and 95% confidence interval (CI) were calculated. Significant *P* value is < 0.05.

## Results

The studied groups showed no statistically significant difference in age and gender. Significant differences were found in median TSH (within reference range) (*p* = 0.013), TG (*p* = 0.008), TC/HDL (*p* = 0.016) and TGs/HDL (*p* = 0.009), but no significant differences were found in the other studied parameters among the studied groups. (Table 1)

**Table 1:** Comparison of anthropometric, clinical and biochemical parameters between studied groups

			Non obese DS *N* = 50	Overweight DS *N* = 24	obese DS *N* = 26	*P* value	P1	P2	P3
Age (years)		Median (min-max)	6 (3.5–16)	**10 (3.5–16)**	**7.8 (3.5–14)**	**0.148** ^K^			
Gender	**Male**	N (%)	29 (58)	**12 (50)**	**16 (61.5)**	**0.698** ^Ȼ^			
	**Female**	N (%)	21 (42)	**12 (50)**	**10 (38.5)**				
T3 (ng/dl)		Mean ± SD	150.4 ± 33.4	**153.9 ± 27.5**	**142.6 ± 27.8**	**0.399** ^A^			
T4 (ug/dl)		Median (min-max)	10.1(4.5–13.1)	**9.7 (1.2–13 )**	**8.5 (5.1–12.3)**	**0.179** ^K^			
TSH (uIU/ml)		Median (min-max)	3.2 (0.5–5.9)	**2.1 (0.6–3.9)**	**2.2 (0.4–3.8)**	0.013 ^K^	**0.05**	**0.049**	1
TC (mg/dl)		Mean ± SD	125.3 ± 37.5	**134.2 ± 31.9**	**135.5 ± 28.4**	**0.369** ^A^			
TGs (mg/dl)		Median (min-max)	94 (9–275)	**129.5 (58–240)**	**118 (67–256)**	0.008 ^K^	0.028	**0.043**	1
HDL-C (mg/dl)		Median (min-max)	33 (15–67)	**27 ( 20–47)**	**32 (20–75)**	**0.092** ^K^			
LDL-C (mg/dl)		Mean ± SD	70.8 ± 22.3	**77.3 ± 25.5**	**72.1 ± 19.5**	**0.611** ^A^			
Non-HDL-C		Median (min-max)	96.5 (35–158)	**97.5 ( 54–156)**	**106.5 (62–163)**	**0.109** ^K^			
TC/HDL		Median (min-max)	3.6 (2.02–6.85)	**4.5 ( 3–8.1)**	**4.3 (2.4–7.8)**	0.016 ^K^	**0.095**	0.034	**1**
TGs/HDL		Median (min-max)	3.4 (0.4–10.6)	**4.5 ( 1.9–9.1)**	**3.9 (1.6–10)**	0.009 ^K^	0.012	0.13	1

A; One way ANOVA test, K; Kruskal Wallis Test; Ȼ; Chi square test, *P* value; comparison between 3 groups, P1; Bonferroni adjusted *P* value between NOD and overweight DS, P2; Bonferroni adjusted *P* value between NOD and OD, P3; Bonferroni adjusted *P* value between OD and overweight DS

This sample of individuals was selected from the population in Dakahleya Governorate in Lower Egypt, with negative consanguinity (random mating). FTO genotypes in NODS group were in HW equilibrium (*p* = 0.64). Our study demonstrated that GT, TT, GT + TT genotypes of FTO rs17917449 and the T allele showed significantly higher frequencies in overweight compared to NODS (p 0.025, 0.033, 0.017, 0.027; OR 4.667, 10.5, 5.069, 2.226*)*. While GT and GT + TT genotypes and the T allele showed significantly higher frequencies in ODS individuals compared to NODS (P 0,036, 0.025, 0.0019; OR, 3.694, 3.983, 3.273). There was no statistically significant difference between overweight and ODS children in this study as regarding FTO genotypes (*p* = 0.955) and alleles (*p* = 0.848). By combining overweight DS and ODS groups and comparing them against the NODS group, there were increased frequencies of the GT, TT, GT + TT genotypes of FTO rs17917449 and the T allele in the overweight/ODS, with a higher risk of developing obesity compared to the NODS groups (*p* 0.005, 0.018, 0.0027, 0.0099; *OR* 4.111, 9.0, 4.448, 2.139) (Table 2).

**Table 2:** Comparison of FTO genotypes and alleles between NODS, overweight DS and ODS groups

	NODS *n* = 50 *N* (%)	Overweight DS *n* = 24 *N* (%)	ODS *n* = 26 *N* (%)	Overweight /ODS *n* = 50 *N* (%)	OR (95% CI)		
					Overweight DS VS NODS	ODS VS NODS	ODS/Overweight DS VS NODS
**GG**	21 (42)	3 (12.5)	4 (15.4)	7 (14)	Reference		
**GT**	27 (54)	18 (75)	19 (73.1)	37 (74)	**4.667** (1.211–17.979) P **= 0.025**	**3.694** (1.091–12.51) P **= 0.036**	**4.111** (1.529–11.051) *P* = 0.005
**TT**	2 (4)	3 (12.5)	3 (11.5)	6 (12)	**10.5** (1.211–91.03) P **= 0.033**	7.875 (0.979–63.312) P **=** 0.052	**9.0** (1.466–55.248) *P* = 0.018
**GT + TT (Dominant model)**	29 (58)	21 (87.5)	22 (84.6)	43 (86)	**5.069** (1.336–19.238) P **= 0.017**	**3.983** (1.194–13.28) P **= 0.025**	**4.4483** (1.675–11.811) *P* = 0.0027
**G**	69 (69)	24 (50)	27 (51.9)	51 (51)	Reference		
**T**	31 (31)	24 (50)	25 (48.1)	49 (49)	**2.226** (1.0978–4.513) P **= 0.027**	**3.273** (1.549–6.914) P **= 0.0019**	**2.139** (1.201–3.809) *P* = 0.0099

OR odds ratio; CI, confidence interval

Analysis of the clinical and laboratory variables among FTO rs17917449 genotypes in DS children revealed no significant differences in age and gender. T allele-containing genotypes showed significantly higher median BMI-centile and TGs compared to the GG genotype. Additionally, a significantly lower median TSH level (within reference range) was observed in the GT genotype compared to the GG genotype (Table 3). In NODS children, median BMI-centiles were significantly higher in the TT genotype than the GG (*p* = 0.038), while HC, head circumference, MAC and WC showed no significant differences between FTO genotypes. Significant differences were observed in mean TC and median TGs levels between FTO genotypes (*P* = 0.016, 0.011). Specifically, mean TC levels were significantly higher in the TT compared to both GG (*P* = 0.022) and GT (*P* = 0.025) genotypes, and the GG genotype compared to the GT genotype (*P* = 0.041). Moreover, the combined (GT + TT) genotypes showed significantly higher mean TC and median TGs levels compared to the GG genotype. In overweight/ODS children, no statistically significant differences were found in anthropometric data according to FTO genotypes (data not shown).

**Table 3:** Comparison of laboratory data according to FTO genotypes in DS children

			GG *N* = 28	GT *N* = 64	TT *N* = 8	GT + TT N = 72	*P*	P1	Post hoc test *P* value		
									GG vs. GT	GG vs. TT	GT vs. TT
Age (years)	Median (min-max)		8 (3.5–16)	6 (3.5–15)	10 (3.5–16)	6.3 (3.5–16)	0.057^K^	0.115^U^			
Gender	Male	N (%)	15 (53.6)	37 (57.8)	5 (62)	42 (58.3)	0.882^**Ȼ**^	0.666 ^**Ȼ**^			
	Female	N (%)	13 (46.4)	27 (42.2)	3 (37.5)	30 (41.7)					
BMI-centile	Median (min- max)		74 (20–98)	92 (54–99)	93.5 (76–96)	92.5 (54–99)	**0.001** ^K^	**< 0.001**^**U**^	0.002	**0.015**	1
HC (cm)	Mean ± SD		49.5 ± 2.9	49.7 ± 3.2	51.5 ± 2.9	49.9 ± 3.2	0.248^A^	0.536 ^**T**^			
A.C (cm)	Median (min- max)		28 (15–46)	29 (15–47)	34.5 (20–49)	30.5 (15–49)	0.104^K^	0.093^U^			
W.C (cm)	Median (min- max)		62 (47–88)	58.5 (47–86)	65.5 (48–87)	59 (47–87)	0.536^K^	0.960^U^			
Head circumference (cm)	Median (min- max)		47.5 (43–56)	47.5 (41–56)	49 (42–54)	48 (41–56)	0.453^K^	0.723^U^			
T3 (ng/dl)	Mean ± SD		146.70 ± 33.642	150.25 ± 30.784	149.88 ± 19.577	150.21 ± 29.642	0.878^A^	0.610^T^			
T4 (ug/dl)	Median (min- max)		9.6 (4.5–12.8)	9.8 (4.9–13.1)	8.6 (5.6–12.5)	9.7 (4.9–13.1)	0.750^K^	0.991^U^			
TSH (uIU/ml)	Median (min- max)		3.2 (0.5–5.9)	2.1 (0.4–4.1)	2.3 (1–4.1)	2.3 (1.4.1)	**0.003** ^K^	**0.009**^**U**^	0.024	0.781	1
TC (mg/dl)	Mean ± SD		119.6 ± 30.5	132.8 ± 34.5	145 ± 36.6	134.1 ± 34.7	**0.1**^**A**^	**0.0.055**^**T**^			
TGs(mg/dl)	Median (min- max)		90.5 (9–174)	112.5 (37–256)	193 (67–275)	116.5 (37–275)	**0.002**^K^	**0.004**^**U**^	0.042	**0.003**	0.122
HDL-C(mg/dl)	Median (min- max)		36 (15–58)	29 (16–75)	29 (21–35)	29(16–75)	0.163^K^	0.065^U^			
LDL-C (mg/dl)	Mean ± SD		67.536 ± 21.8792	73.916 ± 27.0161	81.025 ± 31.6391	74.706 ± 27.4114	0.362^A^	0.219^T^			
Non-HDL-C	Median (min- max)		99.5 (35–158)	100.5 (35–163)	85.5 (45–149)	100 (35–163)	0.984^K^	0.930^U^			
TC/HDL-C	Median (min- max)		3.60 (2.21–8.09)	4.1 (2–7.7)	3.7 (2.81–6.75)	4.1 (2.02–7.79)	0.358^K^	0.160^U^			
TGs/HDL-C	Median (min- max)		3.4 (0.8–9.1)	3.9 (0.4–10.6)	3.8 (1.9–8.3)	3.9 (0.4–10.6)	0.353^K^	0.314^U^			

P, comparison between GG, GT, TT; P1, comparison between GT + TT versus GG; T; t test, U; Mann-Whitney test; A: ANOVA test; K: Kruskal Wallis test; Ȼ; Chi square test, **Post hoc test P value is adjusted by Bonferroni Correction**

To predict obesity in individuals with DS, logistic regression analysis was performed using laboratory data and FTO genotypes as covariates. TGs, Non-HDL-C, TC/HDL, TGs/HDL and FTO GT + TT, TT genotypes were significantly associated with overweight and obesity prediction among DS children in univariate analysis. On the other hand, FTO rs17817449 (GT + TT), TT genotypes and TGs were considered independent risk factors for overweight and obesity prediction in DS children in multivariate analysis (Table 4).

**Table 4:** Logistic regression analysis for prediction of overweight or obesity in DS children

	Univariate			Multivariate		
	P	OR	95% CI	P	OR	95% CI
Age	0.071	1.099	0.992–1.218			
Gender	0.840	1.085	0.491–2.395			
T3 (ng/dl)	0.693	0.997	0.985–1.010			
T4 (ug/dl)	0.071	0.846	0.705–1.014			
TSH (uIU/ml)	0.094	0.720	0.708–1.141			
MPV	0.597	1.078	0.817–1.422			
TC (mg/dl)	0.159	1.009	0.997–1.021			
TGs (mg/dl)	**0.008**	1.011	1.003–1.019	**0.04**	**1.317**	**1.00–2.587**
HDL-C (mg/dl)	0.107	0.969	0.933–1.007			
LDL-C (mg/dl)	0.475	1.006	0.990–1.021			
Non HDL-C	**0.031**	1.009	1.001–1.018	0.192	1.009	0.996–1.022
TC/HDL-C	**0.004**	1.349	1.099–1.655	0.929	1.018	0.695–1.490
TGs/HDL-C	**0.003**	1.196	1.061–1.348	0.086	1.164	0.879–1.584
FTO rs17817449 (GT + TT)	**0.002**	2.511	1.401–4.500	**0.018**	**3.406**	**1.236–9.387**
FTO rs17817449 (TT)	**0.014**	3.853	1.321–11.242	**0.019**	**2.248**	**1.143–4.421**

Logistic regression analysis was used, CI; confidence interval; OR; odds ratio

## Discussion

The present case-control study was designed to assess the obsogenic effect of FTO rs17817449 SNP among a group of overweight/ODS vs. NODS children. There is an increased risk of obesity among individuals with DS. Recently DS children have longer life because of improving the medical care. Physicians should gain a better understanding of the possible causes of obesity in DS children and its related comorbidities.

In the present study, there were statistically significant differences in TG, TC/HDL and TGs/HDL among the studied groups. Friedemann et al. [29], revealed that obese children were more likely to have dyslipidaemia than normal-weight ones. Yahia et al. [30, 31], found that ODS exhibited a prominent atherogenic lipid profile compared to matched obese normal control. In Ordonez-Munoz et al. [32], study, there were significant correlations between higher BMI and waist-to-hip ratio with higher TC and TG and lower levels of HDL in DS Spanish adolescents. On the other hand, Adelekan et al. [33], revealed that abnormal lipid profiles were found in healthy children with DS independent of their weight when compared to NODS children. These data revealed an atherogenic lipid profile among overweight/ODS children, increasing their risk of developing metabolic health problems and CVD. Therefore, routine evaluation of lipid profile and specific preventive strategies, such as low-fat diets and increased physical activity, are recommended for DS children.

The FTO gene has been considered as one of the most important genetic factors regulating the body weight and participate in the development of obesity [34]. The current study revealed that FTO rs17817449 TT genotypes were significantly associated with the risk of overweight and obesity in DS children. Overweight/ODS children carrying the TT genotype have more than nine-fold increased risk of developing overweight/obesity when compared to other genotypes [OR: 9.0; *p* = 0.018]. The true carrier frequency (TT) was 3-fold higher in overweight/ODS than NODS (12% vs. 4%), this gave our study adequate probability to find a difference between both groups and indicated that there was significant association between T allele containing genotypes with obsogenicity in DS. In the present study, the T allele of FTO rs17817449 polymorphism was a risk allele (OR: 2.139) for developing overweight/obesity among DS children. Previous studies demonstrated this contribution of FTO rs17817449 SNP with obesity but there was controversy regarding the risk allele of FTO rs17817449 SNP involved in obesity development. Algenabi et al. [35], on obese Iraqi, Fonseca et al. [36], in Brazilian, Moselhy et al. [6], in Saudi obese subjects and Zaki et al. [37], in Egyptian women, agreed with us in that the T allele of FTO rs17817449 might be a predictor of obesity when compared to the G allele. However, other studies performed on adults and children of European ancestry, Greece children, obese Egyptian females, north Indian Punjabi population, Romanian children, Egyptian obese children and Belarusian population reported that carriers of the G allele had increased level of adiposity compared to T allele carriers [23, 34, 38–42].

On the other side, some investigations displayed no association between FTO rs17817449 genotype frequencies with obesity in Mexican [16, 43], Egyptian [12], Oceanic [44], Japanese [45], Chinese [46], African Americans [47], Latin American [48], and Iranian populations [49]. These controversies could be explained by different ethnicities, different sample sizes in many studies and different methods of genotyping.

The biochemical role of the FTO gene in predisposition to obesity has been discussed in many previous studies. Being largely expressed in hypothalamic nuclei, which control the energy balance, hunger and appetite, the FTO gene may play a role in regulating eating behavior and body metabolism [50, 51]. FTO polymorphisms could activate Iroquois-related homeobox genes (IRX3 and IRX5), which cause cells to store energy in individuals carrying the risk allele. Individuals carrying at least one of the FTO risk alleles have increased food intake compared to those who carry two copies of the normal wild-type variant [16]. On the other hand, cells carrying the normal wild-type allele have IRX3 and IRX5 genes switched off, directing the cells to burn energy [52]. So, carriers of normal wild-type allele tend to have lower body weight, BMI and WC. Recent therapies trying to switch off the FTO risk allele could decrease the percentage of DS children who may become obese by promoting energy burning instead of storage [53]. Kampmann et al. [54], explained that FTO SNPs regulate gene expression at the RNA level through catalyzing the demethylation of 3-methylthymine to 3-methyl uracil in RNA and N6-methyl adenosine in nuclear RNA, which participate in the development of obesity, IR and T2DM.

The present study found no significant differences in age and gender according to FTO genotypes in studied DS children. This indicated that there was no sex-based difference in the heritability of obesity among obese children. Consistent with the current study, Duicu et al. [41], did not observe a significant gender interaction between obesity and FTO rs17817449 in children.

In support of the above findings, FTO was found to have a strong relationship with indicators of obesity. The present study examined the effect of this polymorphism on the anthropometric and biochemical variables in the studied groups. In all studied DS cases, individuals with TT and GT genotypes showed significantly higher median BMI centile vs. GG genotype (*P* = 0.015, 0.002). Similar results were found in the NODS group (*p* = 0.022, 0.011), but in the overweight/ODS group, no significant association between TT genotypes and obesity parameters (*p* > 0.05) was found. This may be due to the limited variation in BMI and the low number of TT cases in that group. The current study reported significantly higher TGs levels in individuals with T allele-containing genotypes (*p* = 0.002) in all studied DS cases. As a result of the higher BMI centile in NODS individuals with the TT genotype, those with higher BMI (TT genotype followed by GT genotype), showed higher mean TC and median TGs concentration (*P* = 0.016, 0.011). Meanwhile, no statistically significant difference was found in BMI and other obesity measures in the overweight/ODS group, as all individuals in this group were overweight or obese. Additionally, logistic regression analysis revealed that the FTO rs17817449 GT + TT, TT genotypes and TGs levels were independent risk factors for predicting overweight/obesity in DS children. Previous studies have shown a significant correlation between the FTO rs17817449 polymorphism and serum TG and/or HDL-C levels [36, 55]. This may be mediated by the activation of adipocytokines, which can lead to the deposition of fatty acids in the liver and skeletal muscles, resulting in insulin resistance and obesity. These findings suggest that rs17817449 variant is significantly associated with biochemical indicators of obesity, and may increase risk of metabolic syndrome [35].

Results of the current study confirmed the findings of other studies performed by Zaki et al. [37], , Attia et al. [56], , Moselhy et al. [6], and Avilés et al. [57], , who found signifiant higher values of BMI and WC in TT rs17817449 carriers compared to GG carriers. However, other studies performed on Quebec Family, Greek children, obese Egyptian children, Belarusian population [23, 38, 42, 58] found that individuals harboring the G allele had raised BMI and WC. On the other hand, Duicu et al. [41], found no relation between the FTO rs17817449 SNP and BMI in children. This variation could also be attributed to differences in ethnicity and sample size. Another explanation for the controversy in results could be increased consumption of fried food with saturated fatty acids that could determine or modulate the association between higher BMI and the FTO risk-allele. The role of FTO genetic polymorphisms in the predisposition to obesity development in Egyptian populations requires further investigations, particularly in relation to the epidemiological transition and to calorie-rich food.

## Conclusion

This is one of the first studies conducted on Egyptian DS children, which revealed that the FTO rs17817449 T allele and GT, TT, (GT + TT) genotypes were associated with increased risk of overweight and obesity. However, FTO rs17817449 (GT + TT), TT genotypes, and TGs were considered independent predictors for overweight and obesity in Egyptian DS children.

## Limitations

It is important to highlight the limitation of this study, which was the small sample size and single center. Therefore, further multicenter large-scale studies with a larger number of cases would be useful to confirm the FTO rs17817449 role in overweight/obesity development in DS. Further studies assessing FTO mRNA and protein expression level in DS are necessary for investigating the relation between the presence of this polymorphism and the eating behavior of these children as overconsumption of fats or refined sugars. Investigating more FTO gene SNPs and their haplotyping analysis in DS children is necessary to achieve more conclusive results about the association of FTO genes in Egyptian obese DS children.

## Supplementary Information

Below is the link to the supplementary material.

ESM 1 (DOCX 7.25 MB)

## Data Availability

No datasets were generated or analysed during the current study.

## Abbreviations

BMI: Body mass index
DS: Down syndrome
FTO: Fat mass and obesity associated gene
HDL-C: High density lipoprotein cholesterol
HC: Hip Circumference
LDL-C: Low-density lipoprotein cholesterol
MAC: Mid arm circumference
NODS: Non obese down syndrome
Non-HDL-C: Non-high-density lipoprotein cholesterol
ODS: Obese down syndrome
PCR-RFLP: Restriction fragment length polymorphism method
TC: Total cholesterol
TG: Triglycerides
WC: Waist circumference
