# Supplementary figures and images for: Association between FTO gene polymorphism and obesity in down syndrome children
Source: Eur J Pediatr. 2024 Dec 21;184(1):95. doi: 10.1007/s00431-024-05909-5 (PMC11662052; doi:10.1007/s00431-024-05909-5)

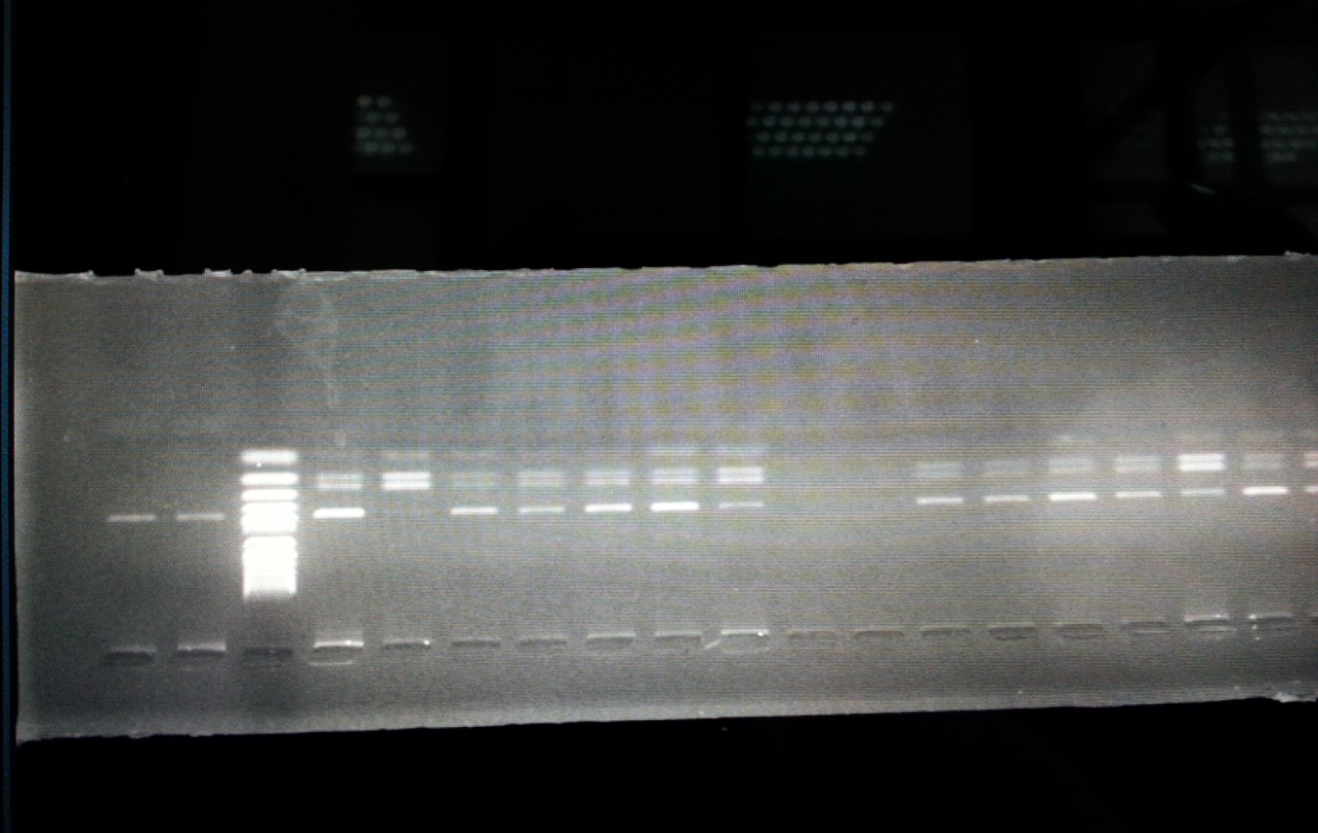


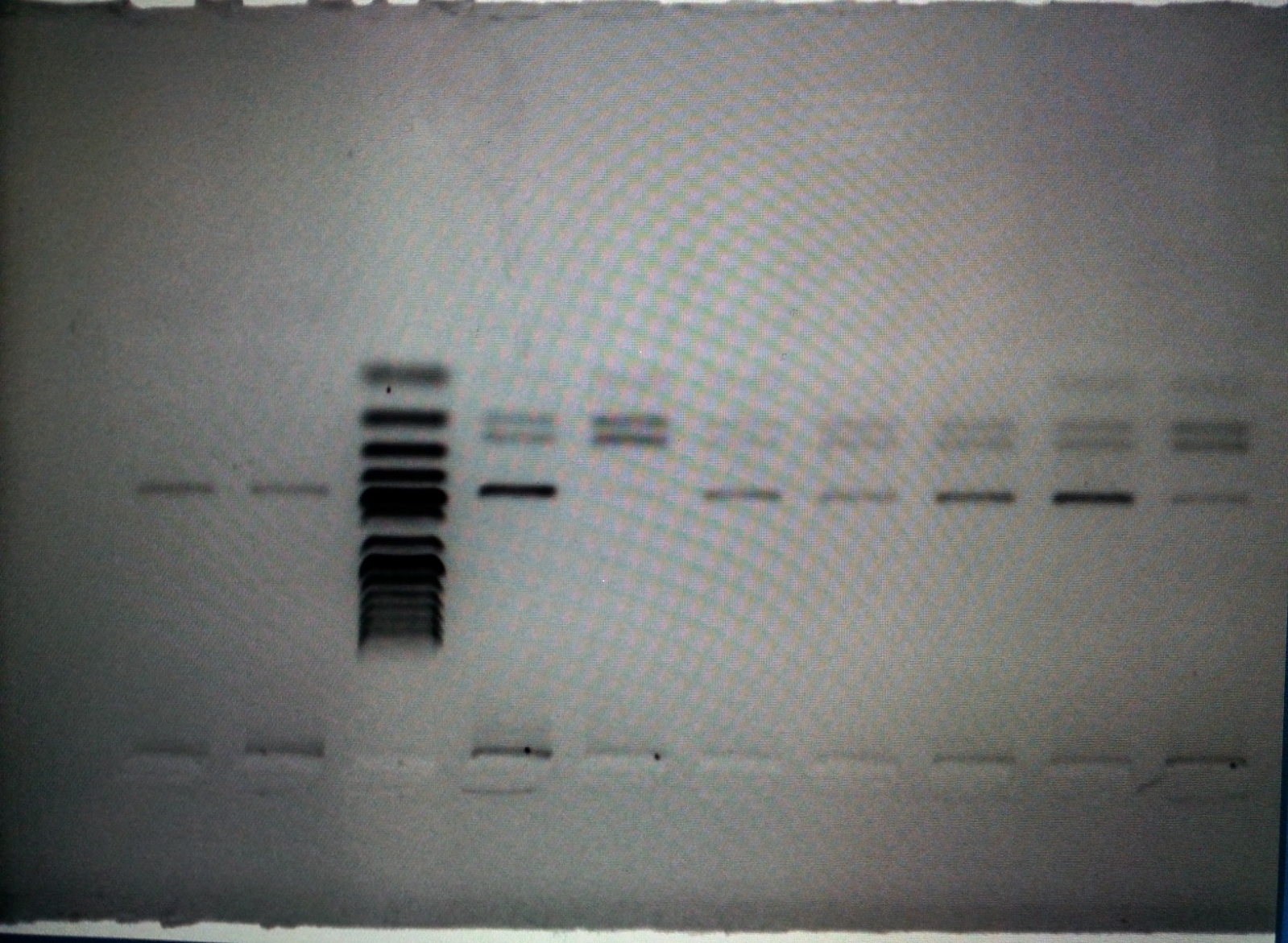


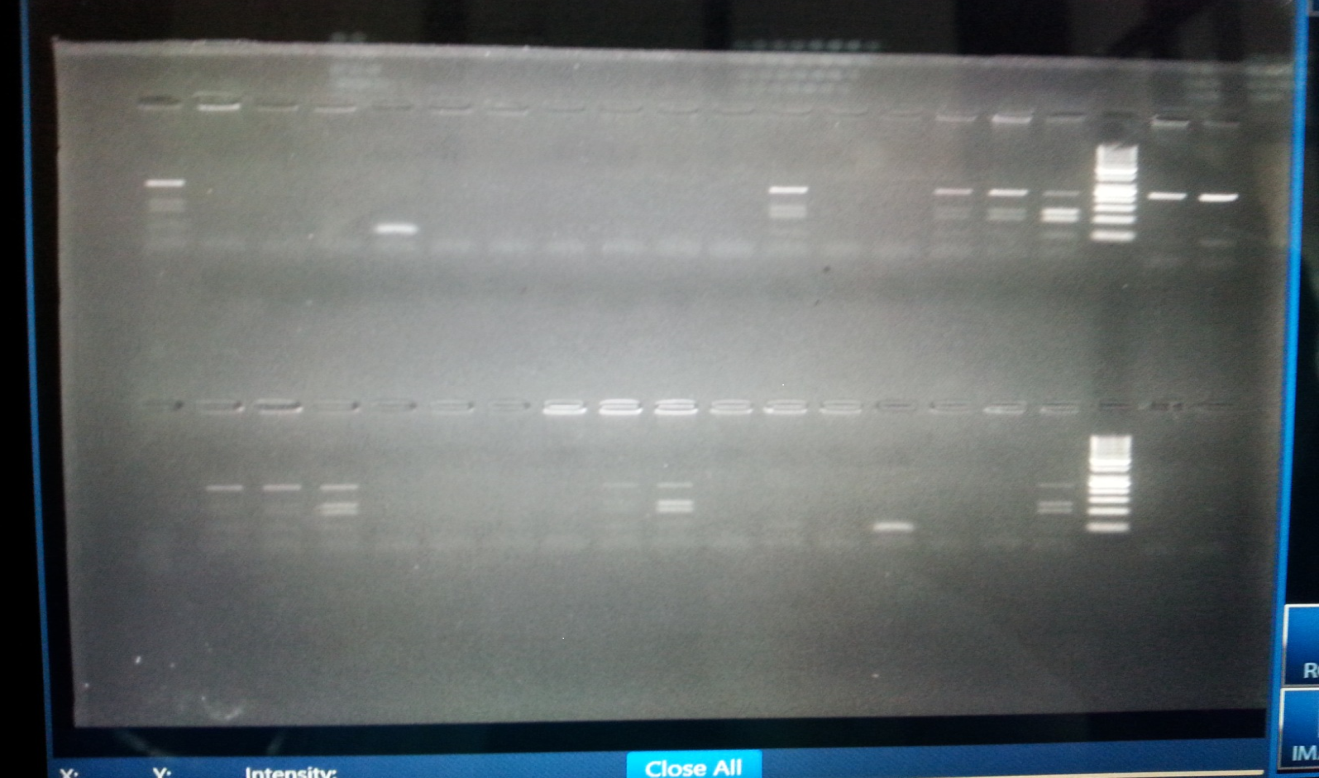


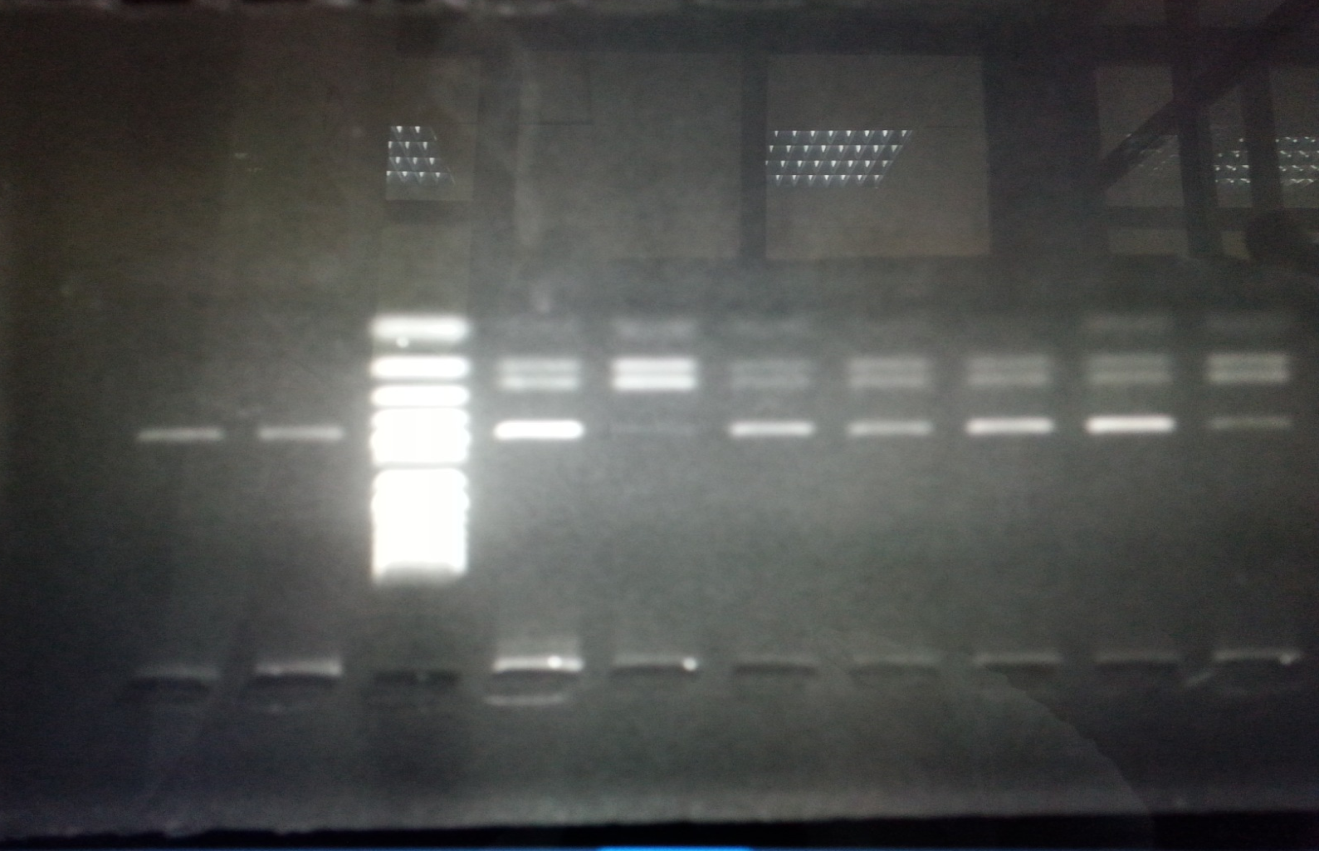

Supplement: Supplementary file 1 — (DOCX 7.25 MB) [file 431_2024_5909_MOESM1_ESM.docx]
